# Comment on Khairul Zaman et al. Eco-Friendly Coagulant versus Industrially Used Coagulants: Identification of Their Coagulation Performance, Mechanism and Optimization in Water Treatment Process. *Int. J. Environ. Res. Public Health* 2021, *18*, 9164

**DOI:** 10.3390/ijerph182212250

**Published:** 2021-11-22

**Authors:** Afia Ivy, Kristian Dubrawski, Caetano Dorea

**Affiliations:** 1 Department of Civil Engineering, University of Victoria, Victoria, BC V8P 5C2, Canada; afiasiddikaivy@gmail.com (A.I.); kdubrawski@uvic.ca (K.D.); 2 Centre for Advanced Materials and Related Technology (CAMTEC), University of Victoria, Victoria, BC V8P 5C2, Canada

**Keywords:** aluminum residuals, Alzheimer’s disease, coagulation, drinking water treatment, natural coagulants, neurotoxicity

## Abstract

In a recent contribution by Zaman and colleagues, a few issues were noted on the justification of their study, which performed a comparative assessment of chitosan as a proposed alternative to aluminum-based coagulants for drinking water treatment applications. We have provided further clarity around such issues, which apply to other studies on the same theme.

Khairul Zaman et al. [1] undertook a comparative assessment of an “eco-friendly” coagulant (i.e., chitosan) and several aluminum-based coagulants. The assessment of a natural coagulant as an alternative to such coagulants for drinking water treatment applications is an interesting topic, and has been the subject of numerous reviews [2,3]. In the study justification, they placed a heavy importance on the reduction of aluminum residuals in finished waters (due to health concerns) as a key reason to consider the naturally derived coagulant chitosan as an alternative to aluminum-based products. We believe that this is perhaps not the strongest argumentation, and point out a factual error in their study justification.

What initially caught our attention was a severe misrepresentation of a previous study of one of our co-authors. It was stated that Bérubé and Dorea [4] “reported a potential link between the neurotoxicity sourced from aluminum and its pathogenesis to Alzheimer’s disease.” That was never the objective or outcome of the study, which evaluated the reduction of aluminum residuals in finished waters in view of several (sometimes competing) conventional water treatment objectives, such as the reductions of turbidity and natural organic matter (NOM)–the precursor of disinfection by-products (DBPs). It was shown that with pH control aluminum, residuals can be minimised to acceptable values. This is in line with World Health Organization guidelines [5], which state that in properly operated systems, aluminum residuals should not be a problem, and that the guideline level of 0.2 mg/L is not health-derived.

Simply put, typically in drinking water treatment the underlying premise is to remove substances from water and not to add to it. So, the minimisation of aluminum residuals is justified. However, the motivation to reduce such residuals could be considered a valid one both as a precautionary measure to any possible health risk (i.e., this has never been conclusively ruled out) and, perhaps more importantly, from an operational standpoint such as increased water turbidity from aluminum hydroxide precipitates [6], reduction of disinfection efficiency [7], and excessive headloss development in filters [8,9] and distribution systems [10]. However, these established issues were not acknowledged by the study in question, and seem to be a common shortcoming of the justification of similar studies.

Zaman et al. correctly pointed out the prevalence of aluminum-based coagulants used in practice for applications such as drinking water treatment. Conversely, natural coagulants have suffered from commercial draw-back [11], and are not widely used in practice. One issue that may be limiting their wider use and that was not investigated or acknowledged by Zaman et al. is the removal of NOM, a key drinking water treatment objective [12] in many contexts. Additionally, we contend that in order to gain mainstream application, alternative coagulants to aluminum-based ones need to provide proven advantages that are either health-based, operational, financial or environmental, etc.–these are significant factors that many natural coagulant studies fail to address.

## References

[1] Khairul Zaman N., Rohani R., Izni Yusoff I., Kamsol M.A., Basiron S.A., Abd. Rashid A.I. (2021). Eco-Friendly Coagulant versus Industrially Used Coagulants: Identification of Their Coagulation Performance, Mechanism and Optimization in Water Treatment Process. Int. J. Environ. Res. Public Health.

[2] Yang R., Li H., Huang M., Yang H., Li A. (2016). A Review on Chitosan-Based Flocculants and Their Applications in Water Treatment. Water Res..

[3] Dorea C.C. (2006). Use of Moringa Spp. Seeds for Coagulation: A Review of a Sustainable Option. Water Supply.

[4] Bérubé D., Dorea C.C. (2008). Optimizing Alum Coagulation for Turbidity, Organics, and Residual Al Reductions. Water Sci. Technol. Water Supply.

[5] World Health Organization (2017). Guidelines for Drinking-Water Quality.

[6] Costello J.J. (1984). Postprecipitation in Distribution Systems. J. Am. Water Works Assoc..

[7] Hoff J. (1977). The Relationship of Turbidity to Disinfection of Potable Water.

[8] Dorea C.C. (2013). Slow Sand Filtration Pre-Treatment with Alum Is Efficient, but Is It Effective?. J. Water Sanit. Hyg. Dev..

[9] Dorea C.C., Clarke B.A. (2006). Chemically Enhanced Gravel Pre-Filtration for Slow Sand Filters: Advantages and Pitfalls. Water Sci. Technol. Water Supply.

[10] Driscoll Charles T., Letterman Raymond D. (1988). Chemistry and Fate of Al(III) in Treated Drinking Water. J. Environ. Eng..

[11] Okoro B.U., Sharifi S., Jesson M.A., Bridgeman J. (2021). Natural Organic Matter (NOM) and Turbidity Removal by Plant-Based Coagulants: A Review. J. Environ. Chem. Eng..

[12] Crittenden J.C., Trussell R.R., Hand D.W., Howe K.J., Tchobanoglous G. (2012). MWH’s Water Treatment: Principles and Design.

